# A Literature Review of Perioperative Outcomes of Robotic Radical Nephrectomy (RRN) Versus Laparoscopic Radical Nephrectomy (LRN) for Renal Cell Carcinoma (RCC)

**DOI:** 10.7759/cureus.49077

**Published:** 2023-11-19

**Authors:** Muhannad Alzamzami, Alsamoal Geirbely, Mohamed B Ahmed, Rabab Osman, Rahi Gandhi, Mahmoud Mohammed, Mohammed Elhadi, Ahmed Kodera

**Affiliations:** 1 Urology, Alexandra Hospital, Worcestershire Acute Hospitals National Health Service (NHS) Trust, Redditch, GBR; 2 Urology, Beaumont Hospital, Dublin, IRL; 3 Urology, Royal Shrewsbury Hospital, Shrewsbury, GBR; 4 Internal Medicine, University Hospital Limerick, Limerick, IRL; 5 Surgical Oncology, South Egypt Cancer Institute, Asyut, EGY; 6 Urology, Worcestershire Acute Hospitals National Health Service (NHS) Trust, Redditch, GBR; 7 Urology, Dudley Group National Health Service (NHS) Hospital, Bromsrgrove, GBR

**Keywords:** laparoscopic radical nephrectomy, renal cancer, radical nephrectomy, robot-assisted laparoscopic nephrectomy, robotic radical nephrectomy

## Abstract

Renal cell carcinoma (RCC) is an adenocarcinoma of the renal cortex. Radical nephrectomy remains the standard of care for managing massive renal tumours. Robotic-assisted radical nephrectomy is an increasing alternative technique to laparoscopic radical nephrectomy (LRN). The da Vinci Surgical System allows for improved dexterity, increased visualisation, tremor filtration and an ergonomic setting to enhance surgeon comfort.

The aim was to compare the perioperative outcomes pertaining to operative time, intraoperative complications, blood loss and length of hospital stay between the robotic and LRN for RCC.

Studies that compared the perioperative findings between robotic radical nephrectomy (RNN) and LRN for RCC were included. The literature review was carried out according to the Cochrane collaboration standards where applicable. Highly sensitive search strategies like MeSH terms and controlled vocabularies were used to identify relevant studies that compare the RNN outcomes to the LRN. Following the literature search, a total of 73 articles were collected, 60 articles were excluded at the stage of reviewing the titles, eight articles were excluded after reading the abstracts, and five articles were included in this paper.

Five studies were included in this analysis, with a total sample size of 1770 patients, 735 were in the robotic arm, and 1035 were in the laparoscopic arm. Generally, there were no differences between both arms in terms of demographic data and age of patients. Closer analysis of the perioperative outcomes did not reveal significant differences between the two groups related to the estimated blood loss, length of hospital stay or post-operative complications. The laparoscopic techniques have less operative time than the robotic ones.

RRN is an expanding approach for patients with RCC with some potential technical benefits over laparoscopic ones. RRN is similar to LRN in the perioperative outcomes, with few potential drawbacks of RRN, including higher costs. However, a prospective comparison of RRN with LRN in many cases at multiple centres with long-term oncological results best illustrates the status of RRN versus LRN.

## Introduction and background

Renal cell carcinoma (RCC) is a neoplasm that originated from the proximal convoluted tubules of the kidney (PCT), mostly solid in nature, with one-quarter of the tumours containing cysts. Areas of necrosis and haemorrhage are common pathological features. RCC comprises different histological subtypes: Clear cell (cRCC), papillary (pRCC), chromophobe (chRCC), collecting duct (Bellini) and medullary cell. Grading is from 1 to 4 using the Fuhrman system, with 1 being well differentiated and 4 being poorly differentiated. Staging follows the Tumour, Node, Metastasis (TNM) classification, with the most up-to-date is the 2017 TNM classification system [1].

The presentation of RCC is mainly an incidental finding of renal mass on imaging such as US and CT scans. The classic triad of flank pain, haematuria and palpable abdominal mass is not commonly seen and is usually associated with advanced disease and poor prognosis. The peak incidence of RCC occurs between 60 and 70 years of age, with a 3:2 male-to-female ratio. Paraneoplastic syndromes are found in approximately 30% of patients with symptomatic RCCs [2].

Radical nephrectomy remains the standard of care for managing massive renal tumours (stage T3 and above or complex renal tumour anatomy). The start of the laparoscopic approach has enabled great benefits over the open approach, mainly in terms of lower perioperative morbidity and faster post-operative recovery. Nonetheless, the open approach has remained preferred for more extensive, locally advanced tumours with venous extension. Over the past decade, robotic surgery has been increasingly adopted in many urologic procedures, including radical nephrectomy, as it can facilitate the surgeon in overcoming particular technical challenges and because of the steep learning curve of standard laparoscopy [3].

The surgical technique typically removes the entire kidney, the fat surrounding it, and sometimes the adrenal gland and lymph nodes. Although studies have sought to evaluate robotic radical nephrectomy (RRN), only some have characterised and compared this technique with the established laparoscopic radical nephrectomy (LRN) [3].

The da Vinci Surgical System continues to permeate all laparoscopic procedures and allows for improved dexterity, increased visualisation, tremor filtration, and an ergonomic setting to enhance surgeon comfort. Although multiple studies have compared open and LRN approaches, few have comparatively investigated robotic and laparoscopic techniques. Over the past decade, robotic technology has been adopted mainly for those major urologic procedures requiring “reconstructive” steps, primarily radical prostatectomy, partial nephrectomy and pyeloplasty. On the other hand, its implementation for purely extirpative procedures, such as radical nephrectomy or adrenalectomy, has remained more limited, given a debatable advantage over standard laparoscopy and concerns of higher costs [3].

The objective is to compare the perioperative outcomes regarding operative time, intraoperative complications, blood loss and length of hospital stay between the RNN and LRN for RCC [4].

## Review

Studies that compared the perioperative findings between RRN and LRN for RCC were considered. Other indications of nephrectomies are excluded from the review as they might increase the risk of selection bias in the study.

The primary outcomes were to compare the perioperative outcomes in terms of operative time, intraoperative complications, blood loss and length of stay between the RRN and LRN for RCC. The secondary outcomes were to compare the oncological outcomes between the RRN and LRN for RCC.

The literature review was conducted according to the Cochrane collaboration standards where applicable. Highly sensitive search strategies like MeSH terms and controlled vocabularies were used to identify relevant studies that compare the RRN outcomes to the LRN. Electronic databases like MEDLINE, EMBASE, and other appropriate resources from the European Association of Urology were sought for studies (Table 1).

**Table 1:** Search strategy

Electronic searches	The advanced search criteria in MEDLINE were used to look for the studies with the terms "robotic radical Nephrectomy", "robot-assisted laparoscopic Nephrectomy", "radical Nephrectomy, “Renal Cancer'', using the Building block technique 1 AND 2 AND 3 AND 4
Concept 1	"Robotics"[Mesh] OR “Robotic Surgery*”[tw] OR “Robotic approach*”[tw]
Concept 2	"Nephrectomy"[Mesh] OR “Radical nephrectomy*”[tw] OR “Kidney removal*”[tw] NOT (partial nephrectomy OR Donor nephrectomy)
Concept 3	"Kidney Neoplasms"[Mesh] OR “Renal Cancer*” [tw] OR “Kidney cancer*” [tw] OR “Renal neoplasm*” [tw]
Concept 4	(("Nephrectomy"[Mesh]) AND ("Laparoscopy"[Mesh]) OR "Hand-Assisted Laparoscopy"[Mesh]) NOT (“Donor nephrectomy*” OR “Partial Nephrectomy”)

The study was conducted according to the Cochrane standards where applicable to assess, analyse and sort the studies into included and excluded ones. Following the literature search, a total of 73 articles were collected; 60 articles were excluded after reviewing the titles, eight were excluded after reading the abstracts, and five were included in this paper.

The primary data related to the objectives of this paper were extracted. The following variables were extracted: authors, year of publication, DOI, sample sizes of each arm of the trials, participants’ mean age, mean operative time for each component and its standard deviations, mean blood losses for each arm and its standard deviations, mean hospital stay for each arm and its standard deviations, mean complication rate for each component. The data of interest was copied to the RevMan (Review Manager Software, v. 5.3, Cochrane Collaboration, Oxford, UK) data analysis tool to create the tables and figures shown in this paper.

The evaluation of bias assessment was done using the Cochrane assessment tool for RCT, which contains the following; Randomization procedure, blinding, selective reporting, and other sources of bias. Two team members assessed the risk of bias and any disagreement resolved by discussion or arbitration from the Mentor. Meticulous screening of the included studies led to the collection of most of the required data. However, on limited occasions, missing data were calculated using the RevMan tool for data and analysis.

All qualified studies were assessed for clinical heterogeneity. Statistical heterogeneity was evaluated using eyeball and Chi-square tests to check if the P value would suggest the presence of heterogeneity. Quantification of heterogeneity was calculated with the I-squared test to assess how much heterogeneity affects the outcome of the study analysis. All the included studies were checked for any significant conflict of interest. Study citations and inclusion in Journals and other publication types might have been considered during the assessment. The final display of the risk of bias is done using the RevMan risk of bias tool.

A predesigned Excel Sheet (Microsoft® Corp., Redmond, USA) was designed and made available for data collection and analysis, including the means, standard deviations and dichotomous outcomes. The Cochrane RevMan is then used for the statistical analysis where relevant and calculation of a few missing data. Dichotomous variables analysis used an odd ratio (OR) with a 95% confidence interval (95% CI). When both arms showed no event, a risk difference with 95% CI is used for analysis. A p-value of less than 0.05 is considered significant; heterogeneity was measured using I2 and X2 tests. No subgroup analysis was done for this review. RRN is a relatively new practice, where the published randomised controlled trials are still relatively few, thus a tiny pooled sample size. RevMan heterogeneity calculation tools did the heterogeneity analysis, and all were included in the result section.

Seventy-three studies were identified from EMBSAE, MEDLINE, Cochrane Library, and the EAU citation and references. The total sample size is 1770 patients, 735 were in the robotic arm, and 1035 were in the laparoscopic arm. Generally, the participant demographics data did not show a significant difference in the five studies selected [3-7]. The mean age was comparable between all studies. The other three studies showed higher male-to-female ratios than Hemal et al. and White et al. The essential characteristics of the studies are displayed in Table 2.

**Table 2:** Basic characteristics

Study	Robotic arm N	Laparoscopic arm N	R mean age	L mean age	Sex M%	Sex F%
Anele 2019 [3]	404	537	62.3	63	69.2	30.8
Hemal 2008 [4]	15	15	50.3	52.7	46.6	53.4
Helmers 2016 [5]	76	243	63	62.1	64.4	35.6
Golombos 2016 [6]	230	230	73.7	74.2	65.2	34.8
White 2011 [7]	10	10	64	64.5	50	50

**Figure 1:**
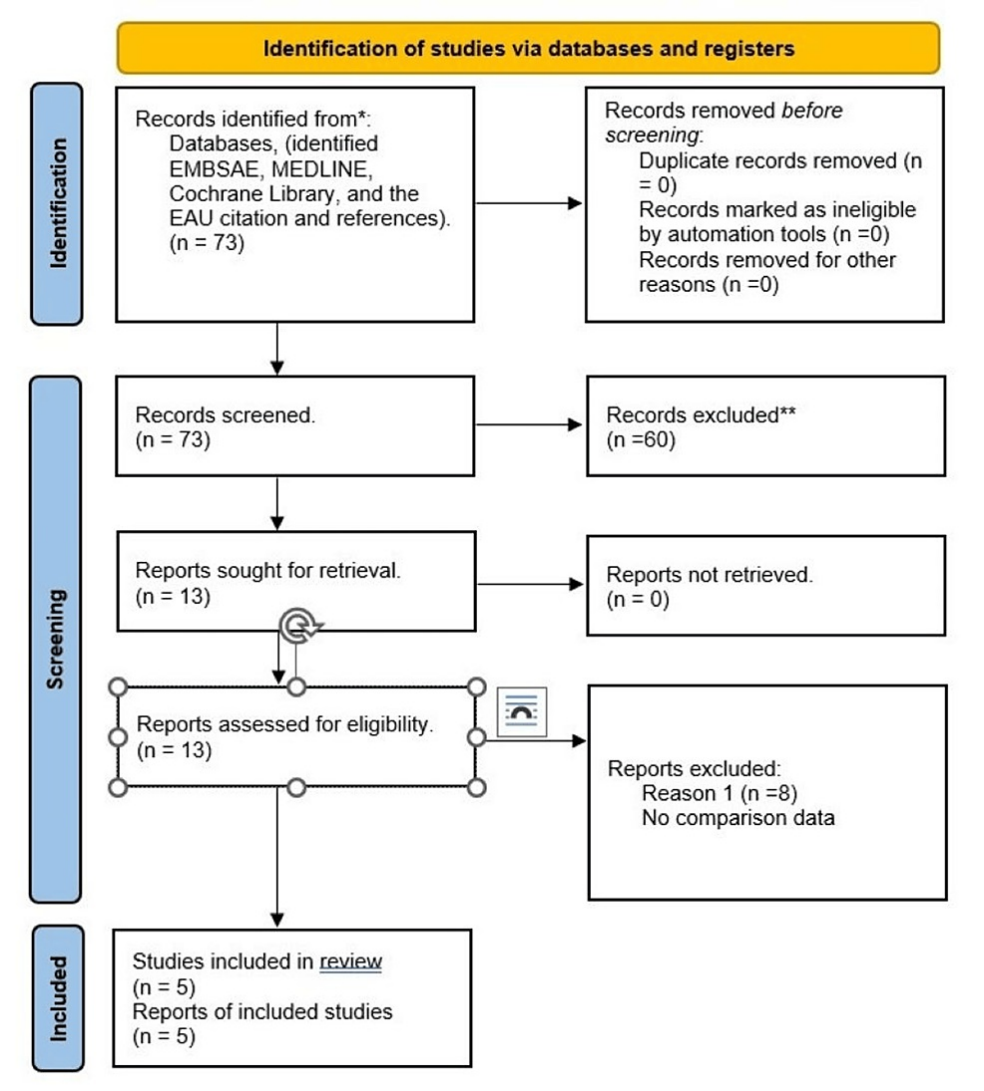
Search methodology

Seventy-three studies were identified from the abovementioned resources. Five studies met the proper criteria for inclusion and were found to serve the purpose of this review. Following the review of the title and abstract of all 73 studies, 60 and eight were excluded, respectively, deemed unsuitable for the review. Propensity-matched, cost-analysis studies and national studies are potential sources of valuable data. However, including those studies could add to the sample size and widen this paper’s scope. The cost analysis studies could add to the purpose of this review. However, they were excluded from this review for the sake of the available resources and time (Figure 1).

**Figure 2:**
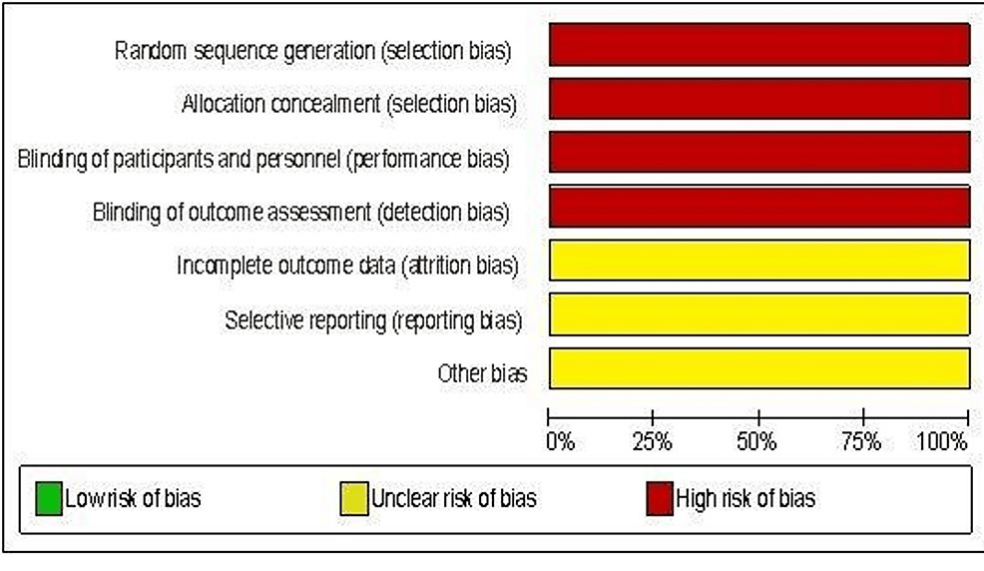
Summary of bias assessment for included studies

**Figure 3:**
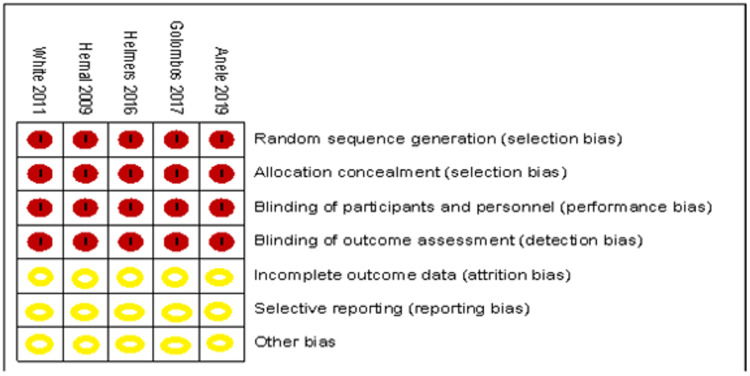
Summary of bias assessment for included studies Anele 2019 [3], Hemal 2008 [4], Helmers 2016 [5], Golombos 2016 [6], White 2011 [7]

All the included studies showed evidence of allocation concealment/randomisation (selection bias) and proof of binding of participants/surgeons (performance bias), which can be attributed to the nature of the surgical intervention and the ethical implication for blinding of such treatment. Also, the selected studies showed evidence of binding outcome assessment (detection bias). It wasn’t clear for all of the included studies whether they possessed features of incomplete outcome data (attrition bias), selective reporting (reporting bias) or other types of bias. Other sources of bias cannot be excluded, and it could be technically challenging to follow every risk of bias. For instance, the surgeon’s experience with the robot, the theatre set-up, and personnel familiarity with the robot have not been reported in any trials. The author of this paper argues that the experience factor and the hospital set-up are essential factors when reporting the robotic outcome, especially when the standard technique requires a relatively simple standard theatre and logistic setup. But generally, the included studies showed signs of good quality and no obvious significant concerns about the conduct of these studies. A summary of the bias assessment was done with the RevMan risk of bias table, shown below in Figures 2, 3.

**Figure 4:**
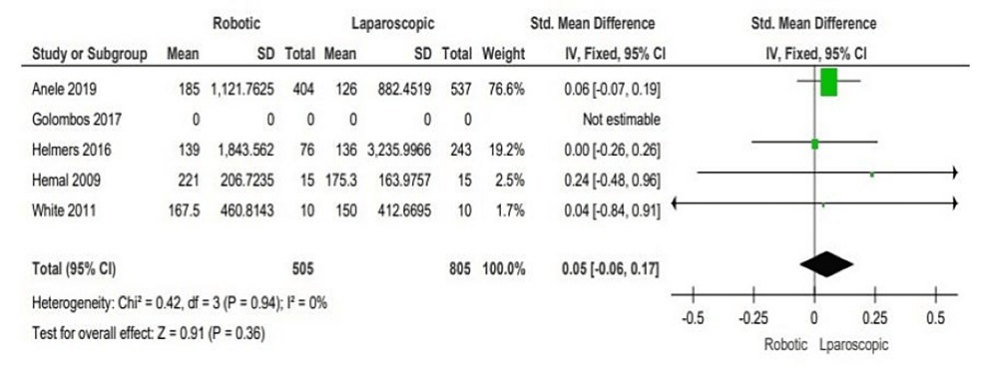
Analysis of the operative time outcomes Anele 2019 [3], Hemal 2008 [4], Helmers 2016 [5], Golombos 2016 [6], White 2011 [7]

Extraction of the data from all of the included studies [3-7] showed shorter operative time in the laparoscopic arms [3,4,7] (weighted mean difference (WMD): 0.05, 95% CI: -0.06 to 0.17; P = 0.36), only Helmers et al. [5] showed no significant differences in the operative time between both arms. By an abstract look at the forest plot, the laparoscopic techniques seem to have less operative time than the robotic counterparts; Figure 4 below shows the operative time outcomes.

**Figure 5:**
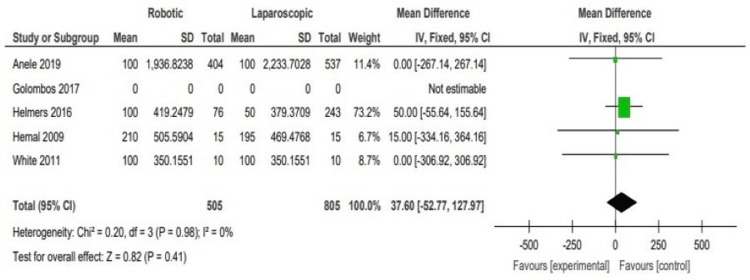
Analysis of the estimated blood loss (EBL) Anele 2019 [3], Hemal 2008 [4], Helmers 2016 [5], Golombos 2016 [6], White 2011 [7]

The selected studies reported no vast difference in the estimated blood loss (EBL) between the robotic and laparoscopic arms (WMD: 37.60, 95% CI: -52.77 to 127.97; P = 0.41). Overall, the forest plot in analysis (Figure 5) shows the results from all the studies with no significant difference between the experimental group (robotic arm) and the control group (laparoscopic arm), with all studies’ mean differences lying relatively close to the line of no difference.

**Figure 6:**
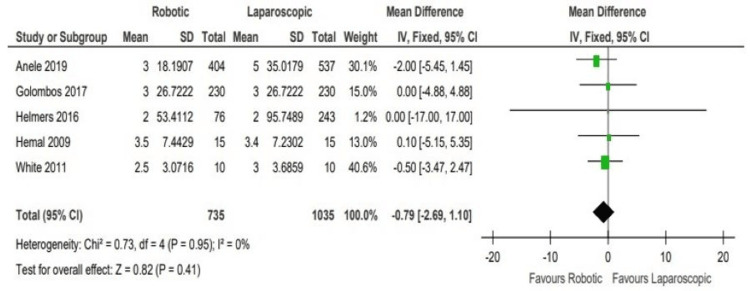
Analysis of the length of stay (LOS) Anele 2019 [3], Hemal 2008 [4], Helmers 2016 [5], Golombos 2016 [6], White 2011 [7]

No significant difference was found regarding the length of stay (LOS), where all the selected studies were precisely on the line of no difference, apart from the Anele et al. study [1], which was also relatively close to the line of no difference (WMD: -0.79, 95% CI: -2.69 to 1.10; P = 0.41) (Figure 6).

**Figure 7:**
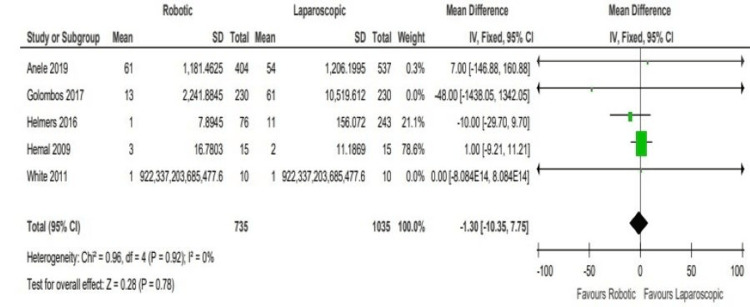
Analysis of post-operative complications Anele 2019 [3], Hemal 2008 [4], Helmers 2016 [5], Golombos 2016 [6], White 2011 [7]

No significant difference was found between the two comparative arms in any of the selected studies regarding post-operative complications. We can appreciate that all four studies lying either on the line of no difference or close to it, except for Golombos et al. [6], which was away from the line and favours the robotic arm (WMD: -1.30, 95% CI: -10.35 to 7.75; P = 0.18). Most studies specified the complications period within the 90 days following the surgery (Figure 7).

During the last decade, LRN has been considered the gold standard treatment in patients with RCC. However, laparoscopic urologic surgeries are being practised by a limited group of urologists due to the limited exposure during urological residencies [8]. Thus, more attention is needed for patients with RCC as the oncological features are vital during the surgery. Therefore, as the need arises to progress in surgical learning and skills, many alternative modalities to perform the radical nephrectomy have been implemented, like hand and robotic assistance. The robotic-assisted laparoscopic surgery is an excellent assist to traditional laparoscopy, as considered one of the modalities of minimally invasive surgery; it helped shorten the learning curve and technical challenges of laparoscopy but with serious consideration of the cost [9].

RRN is deemed one of the valid options for patients requiring the removal of the entire kidney due to RCC [10]. However, some queries related to cost and time demands were raised using robotic surgery when performing radical nephrectomies [11]. Nevertheless, more studies are needed to check for further benefits from RRN, as only a few comparisons are available between RRN and LRN [4].

The da Vinci robot system has various technical advantages as it provides wide degrees of freedom of movement and 3-division, which helps perform precise dissections in the laparoscopic environment [10]. In contrast, robotic assistance surgery has some disadvantages like high cost, more time for preparation and performing the procedure, special training on how you can use it and the need for an experienced bedside surgical assistant to play essential tasks like retraction, suction, changing of instruments and application of clips on vessels, which in some instance can create significant bleeding and increase the rate of conversion to open surgery [4].

Many studies compared open and laparoscopic techniques when performing radical nephrectomies, but only a few studies compared the robotic versus the laparoscopic approaches [12,13]. Because of the high costs and other disadvantages mentioned in the literature, we aimed to determine if RRN is superior to LRN in terms of measurable perioperative outcomes.

We found shorter operative time in the laparoscopic arms in three out of five studies [3,4,7] (WMD: 0.05, 95% CI: -0.06 to 0.17; P = 0.36), only one study [5] showed no significant differences in the operative time between the robotic and laparoscopic arms. Golombos et al. didn’t include the operative time outcome in their study. No study reported the details of each part of the surgical procedure. The forest plot shows that the laparoscopic arm has a shorter operative time than the robotic arm. This can be explained by the longer time needed for the operative set-up for robotic surgery; this time can be prolonged with the less experienced surgical team with the knowledge of the robotic troubleshooting setup. We believe this longer operative time might be shorter in the future with the mechanical training and the robotic availability become more pronounced, not to forget that the laparoscopic approach was used earlier than the robotic approach; however, more studies are needed to reach such a result.

Looking at the EBL, we can highlight that the selected studies reported no considerable difference in the EBL between the robotic and laparoscopic arms (WMD: 37.60, 95% CI: -52.77 to 127.97; P = 0.41). Again, Golombos et al. should have mentioned the EBL in their study. An overall look at the forest plot in Figure 4 shows that the EBL is similar between both groups; a primary reason for that is the use of port access when performing robotic or laparoscopic surgery, which is associated with less need for surgical retractors-furthermore, better visibility and the ergonomic accessibility offered by the new technology.

Regarding the length of hospital stay when comparing both modalities, we can appreciate that there is no significant difference among both arms, where all the selected studies lie on or near the line of no difference, as evident in Figure 5 (WMD: -0.79, 95% CI: -2.69 to 1.10; P = 0.41).

A careful look at the post-operative complications following the robotic and laparoscopic nephrectomy among the selected studies within 90 days from the surgery revealed no significant difference when performing either modality, where all four studies were on the line of no difference except one study [3], which however, still there was no statistical difference as shown in Figure 6.

RRN is gaining popularity as an alternative technique to LRN, and the latter is considered the actual gold standard for treating clinically localised RCC. Limited evidence is currently available on the role of robotics in the field of radical nephrectomy [14].

Helmers et al. reported that RRN cases were more likely to require conversion to alternative approaches than LRN due to bleeding, dense adhesions and unamenable tumour characteristics. Such conversion was suggested to be linked to the learning curve associated with RRN. In addition, Helmers and colleagues reported that RRN cases were more likely to include lymph node dissection (LND) [5], which was also highlighted by Ambani et al. [15].

In the large multi-institutional study by Anele and colleagues [3], patients with higher BMI following RRN were linked to a higher rate of positive margins and higher pathologic grade and stage, highlighting the use of robotic modality to manage more complex tumours. Leow et al. concluded the same remark during their comparative meta-analysis of nearly 5000 patients [16]. The robotic approach also demonstrated the feasibility and safety of challenging procedures such as inferior vena cava thrombectomy.

Golombos et al. alluded to some potential technical benefits of the robotic approach over the laparoscopic one, which provides better perioperative outcomes for RRN. For instance, the surgeon can access the renal vessels with the articulated clip applier at angles not achievable with a conventional laparoscopic clip. Furthermore, the endo-wrist technology may allow for more uncomplicated ligation of renal vessels [6]. Veccia et al. have further mentioned that robotic nephroureterectomy appears to offer the advantages of a minimally invasive approach without impairing the oncological outcomes [17].

Cost analysis is an essential determinant factor, Kates et al. used HSCRC data for all RN cases performed in Maryland between 2008 and 2012; they found a total hospital charge premium of $5,111 for RRN compared to LRN ($23,391 for RRN and $18,280 for LRN). Similar observation by Yang et al. through a retrospective cost analysis of 24,312 RN cases from the National Inpatient Sample. They reported that total hospital costs for RRN were $15,149 compared to $11,735 for LRN. Helmers and colleagues reported a lower charge premium of $1352 for RRN, which is likely multifactorial and reflects the thoughtful use of disposables in the operating room and a mature clinical pathway for robotic cases [5]. A prospective comparison of RRN with LRN in many cases at multiple centres with long-term oncological results best illustrates the status of RRN versus LRN. Concerning the learning curve of these minimally invasive procedures, surgeons seem to have a longer learning curve with the laparoscopic approach in comparison to the robotic approach as highlighted by Guerrero et al. [18]. Gab Jeong et al. mentioned that RRN was linked to longer operative time and higher hospital cost when compared to LRN, but not associated with major post-operative complications [19]. Further studies demonstrated that radical nephrectomy can be safely performed by robotic approach without significant difference in perioperative complication rates and can be a viable alternative for performing radical nephrectomy [10,20].

To highlight, five studies were included in this analysis [3-7], with a total sample size of 1770 patients, 735 were in the robotic arm, and 1035 were in the laparoscopic arm. Generally, there were no differences between both arms in terms of demographic data and age of patients. Closer analysis of the perioperative outcomes did not reveal significant differences between the two groups related to the EBL, length of hospital stay or post-operative complications. The laparoscopic techniques have less operative time than the robotic ones.

This paper is a review of the available literature on RRN versus LRN; to the best abilities of the author related to time and resources consideration. RevMan statistical resources analysed the results, and the Cochrane outlines of conducting a systemic review have been followed as much as possible. Although the non-randomization pattern of the selected studies could intuitively bring the level of evidence order for this paper down, the amount of evidence shown in this review could form a reasonable judgment when comparing the RRN to the LRN approach.

The results shown might not be conclusive and include all aspects of radical nephrectomy when comparing the robotic with the laparoscopic approach. The exclusion of studies in other languages could create an obvious risk of bias. In addition, all of the included studies showed evidence of allocation concealment and randomisation (selection bias).

## Conclusions

RRN is an expanding approach for patients with RCC with some potential technical benefits of better ergonomics for the surgeons over laparoscopic one. RRN is similar to LRN in the perioperative outcomes, with few potential drawbacks of RRN, including higher costs. However, a prospective comparison of RRN with LRN in many cases at multiple centres with long-term oncological results best illustrates the status of RRN versus LRN.

RRN is an increasingly utilised alternative to LRN. Despite its late start, it seems to replace the conventional radical nephrectomy approach. Our review did not show a significant difference between the robotic and the laparoscopic modalities in the perioperative outcomes mentioned earlier. However, some technical benefits favour the robotic approach. Further, blinded randomised controlled trials are needed to demonstrate the extension between the two approaches. Furthermore, other information like the surgeon’s experience and centre facilities should be considered.

This paper can serve as an addition to the scope of robotic versus laparoscopic comparative studies when dealing with radical nephrectomies. Additional factors like free time and funding are required to elaborate further details about the robotic approach. However, with time, the robotic approach will expand and reveal an accurate estimation of perioperative outcomes related directly to the patients, surgeons and stakeholders.
